# Transcriptome analysis of PK-15 cells in innate immune response to porcine deltacoronavirus infection

**DOI:** 10.1371/journal.pone.0223177

**Published:** 2019-10-01

**Authors:** Shan Jiang, Fuqiang Li, Xiuli Li, Lili Wang, Li Zhang, Chao Lu, Li Zheng, Minghua Yan

**Affiliations:** 1 Tianjin Institute of Animal Husbandry and Veterinary Medicine, Tianjin Academy of Agricultural Sciences, Tianjin, China; 2 Tianjin Scientific Observation Experiment Station for Veterinary Medicine and Diagnosis Technology, the Ministry of Agriculture of the People`s Republic of China, Tianjin, China; Plum Island Animal Disease Center, UNITED STATES

## Abstract

Porcine deltacoronavirus (PDCoV) is a newly emerged swine enteropathogenic coronavirus affecting pigs of all ages and causing diarrhea problems. Research findings indicate that PDCoV has evolved strategies to escape innate immune response in host cells, but mechanism of PDCoV in innate immune modulation is not well understood. In this study, we report our findings on identifying the alterations of host cell innate immune response affected by PDCoV infection and exploring the gene expression profiles of PK-15 cells at 0, 24, and 36 h PDCoV post infection by RNA sequencing. A total of 3,762 and 560 differentially expressed genes (DEGs) were screened by comparison of uninfected PK-15 cells and infected PK-15 cells at 24 h post infection (hpi) (INF_24h versus NC), and also comparison of infected PK-15 cells between 24 and 36 hpi (INF_36h versus INF_24h), which included 156 and 23 porcine innate immune-related genes in the DEGs of INF_24h versus NC and INF_36h versus INF_24h, respectively. Gene Ontology function classification and Kyoto Encyclopedia of Genes and Genomes signaling pathway enrichment analysis were performed based on the DEGs that exhibited the same expression tendencies with most of the innate immune-associated genes among these PK-15 cell samples described above. The enrichment results indicated that extensive gene functions and signaling pathways including innate immune-associated functions and pathways were affected by PDCoV infection. Particularly, 4 of 5 innate immune signaling pathways, which were primarily affected by PDCoV, played important roles in *I-IFN*’s antiviral function in innate immune response. Additionally, 16 of the host cell endogenous miRNAs were predicted as potential contributors to the modulation of innate immune response affected by PDCoV. Our research findings indicated that the innate immune-associated genes and signaling pathways in PK-15 cells could be modified by the infection of PDCoV, which provides a fundamental foundation for further studies to better understand the mechanism of PDCoV infections, so as to effectively control and prevent PDCoV-induced swine diarrheal disease outbreaks.

## Introduction

Porcine deltacoronavirus (PDCoV) is a newly emerged swine enteropathogenic coronavirus that can cause acute diarrhea, vomiting, dehydration, and mortality in pigs [1, 2]. PDCoV was first discovered in the rectal swab of pigs in Hong Kong and was initially reported in 2012 [2, 3]. In 2014, it appeared in the state of Ohio in the United States, resulted in outbreaks of diarrheal disease of piglets and sows accompanied by 30~40% mortality rates in piglets, and later rapidly spreading to several other states adjacent to Ohio [4, 5]. At present, PDCoV has been identified in multiple countries including Canada, South Korea, Thailand, and China [6–9]. Although a variety of PDCoV detection approaches have been established and effectively used for diagnostic tests, such as one-step quantitative real-time PCR (qRT-PCR) assays and enzyme linked immunosorbent assay (ELISA) [10, 11]. However, there are still no effective treatments and control measures for swine PDCoV infections due to its mechanism of PDCoV infections is mostly remained unknown.

Innate immune response provides an essential early line of host cell defense against viral infection. When a virus invades a host cell, viral pathogen-associated molecular patterns (PAMPs) are recognized by host cell pattern recognition receptors (PRRs), primarily consisting of toll-like receptors (TLRs), retinoic acid-inducible gene (RIG)-I-like receptors (RLRs), nucleotide-binding and oligomerization domain (NOD)-like receptors (NLRs), and cytoplasmic DNA sensors. Subsequently, PRRs stimulate signaling that triggers an innate immune response inducing the production of type I interferons (*I-IFNs*), mainly consisting of several *IFN-α* subtypes and *IFN-β*. *I-IFNs* are the major antiviral cytokines of innate immune response that mediate related responses resulting in the production of *IFN*-stimulated genes (ISGs) in order to control virus replication [12, 13, 14].

To successfully proliferate in host cells, multiple types of coronaviruses have evolved different strategies in order to evade cell innate immune response either by inhibiting the production of *I-IFN* or by disrupting *I-IFN*-involved innate immune signaling pathways. Severe acute respiratory syndrome coronavirus (SARS-CoV) can induce the formation of acute respiratory distress in human beings and has a mortality rate close to 10%. SARS-CoV has gained widespread attention since it first emerged as a huge threat to humans in 2002. In order to explore the pathogenic mechanism of SARS-CoV, various studies have focused on cellular mechanisms, including innate immune response evasion. Kopecky-Bromberg SA *et al*. found that the nucleocapsid (N) protein which encoded by SARS-CoV againsts *IFN-β* production by inhibiting *IRF-3*, the key protein necessary for the expression of *IFN-β* [15]. Furtherly, Hu Y *et al*. found that N protein of SARS-CoV interfers with RIG-I activation and ubiquitination which mediated by tripartite motif protein 25 (TRIM25), resulting in the inhibition of *IFN-β* production which achieve higher SARS-CoV replication in host cells [16]. In addition to SARS-CoV, the N protein of other coronaviruses, such as mouse hepatitis virus (MHV), has also been identified as an innate immunity antagonist. RIG-I and melanoma differentiation gene 5 (MDA5) are both PRRs that can recognize PAMPs and then activate signaling to promote *I-IFN* production. MHV N protein inhibits the synthesis of *IFN-β* by interacting with the protein activator of protein kinase R (PACT), which can bind to RIG-I and MDA5, promoting the production of *IFN-β* [17]. As an avian coronavirus, infectious bronchitis virus (IBV) escapes innate immune response by cleaving the mitochondrial antiviral signaling protein (MAVS), which disrupts downstream of RIG-I and MDA5, antagonizing *IFN-β* synthesis [18].

In terms of PDCoV, there are also several strategies have been evolved to escape innate immune response. PDCoV acts against the innate immune response by impeding the activation of transcription factors *IRF3* and *NF-κB*, both of which are involved in the RIG-I signaling pathway disrupting *IFN-β* production [19]. Nonstructural protein 5 (NSP5), which is encoded by PDCoV, antagonizes *I-IFN* signaling through modulation of the Jak/STAT signaling pathway by cleaving STAT2, resulting in the disruption of ISGs production [20]. The *NF-κB* essential modulator (NEMO) is a critical constituent of the IKK complex (IKK-α, IKK-β, and IKK-γ) involved in downstream of RIG-I and MDA5 signaling, which is necessary for *I-IFN* production. The cleavage of NEMO caused by NSP5 impedes the synthesis of *I-IFN* [21]. In addition to nonstructural proteins, accessory proteins such as NS6 encoded by PDCoV also play an important role in the inhibition of *IFN-β* production by interfering with the binding of RIG-I/MDA5 and PDCoV [22]. However, the alteration of host cell innate immune response affected by PDCoV infection still needs to be explored. In particular, the main innate immune-associated genes and signaling pathways of host cell modulated by PDCoV have not yet been uncovered, which can hinder further discoveries regarding the mechanism of PDCoV infection.

MicroRNAs (miRNAs) are a group of short noncoding RNAs known as gene regulators that can modulate extensive bioprocesses by repressing the expression of their target genes at the translation level and/or mRNA level. It has been reported that the expression of miRNAs in host cells can be changed by multiple coronaviruses, such as severe acute respiratory syndrome coronavirus (SARS-CoV), hepatitis C virus (HCV), porcine epidemic diarrhea virus (PEDV) and transmissible gastroenteritis virus (TGEV) [23, 24, 25, 26]. Some of the host cell endogenous miRNAs have been found to play important roles in the modulation of innate immune response affected by coronavirus. On one hand, host cell-derived miRNAs can enhance the innate immune response to inhibit the replication of coronaviruses. For excample, miRNA-221-5p (miR-221-5p) activates *NF-κB* signaling pathway which leads to the upregulation of *I-IFN* and the downstream ISGs as well as the suppression of PEDV replication [27]. On the other hand, cell endogenous miRNAs also can inhibit innate immune repsonse resulting in coronaviruses innate immune evasion. For example, inositol-requiring enzyme 1 α (*IRE1α*) modulates the decreasing of miR-30a-5p which involved in the signaling pathway of miR-30a-5p/*SOCS*1/3, resulting in the promotion of TGEV’s replication by escaping innate immune response [28]. Interferon stimulatory gene (*IFITM1*) is a target of miR-130a. Knockdown of miR-130a can increase the expression of *IFITM1* resulting in the inhibition of HCV replication, which indicate that miR-130a directly involves in innate immune evasion of HCV [29]. As of yet, however, there has been no research into the host cell endogenous miRNAs which may involve in innate immune response modulation affected by PDCoV infection.

To explore the modulation of innate immune response of host cell affected by PDCoV, this study investigated the global gene expression profiles of PK-15 cells at 0, 24, 36 h post infection (hpi) via RNA sequencing. Subsequently, differentially expressed genes screening, gene function and signaling pathway enrichment analysis, and the prediction of PK-15 cell endogenous miRNAs that may make constributions to the innate immune response modulation affected by PDCoV were further performed. Our study provides a foundation for future systematic explorations of the PDCoV infection mechanism.

## Materials and methods

### PK-15 cell culture

PK-15 cells, which were stored in our cell culture lab in this institute (Tianjin Institute of Animal Husbandry and Veterinary Medicine, Tianjin Academy of Agricultural Sciences, Tianjin, China) and used in our previous research project [30], were used in this study. The PK-15 cells were cultured in Modified Eagle’s Medium (MEM) (HyClone) supplemented with 8% fetal bovine serum (ExCell), and 1% penicillin and streptomycin. Cell culture flasks were held at 37°C in a humidified incubator set at 5% CO_2_.

### PDCoV preparation and inoculation

The PDCoV strain in this study was previously isolated by Swine Disease Research Innovation Lab of our Institute from small intestine specimens of piglets showed diarrheal syndromes [30]. Those piglets were raised in a swine farm in a county under Tianjin municipal administration. This PDCoV strain was propagated in PK-15 cell cultures. MEM supplemented with 1% pancreatin (Sigma-Aldrich) was prepared for PDCoV dilution and cell culture after viral adsorption. PDCoV with a final concentration of 0.1 multiplicity of infection (MOI) was used for adsorption in single-line PK-15 cells for 2 h, followed by replacement with new cell culture medium. At 0, 24, and 36 hpi, cells were collected for RNA isolation.

### Indirect immunofluorescence assay (IFA)

PDCoV infected PK-15 cells with 0.01 multiplicity of infection (MOI) and uninfected PK-15 cells in 96-well plates were washed twice with phosphate-buffered saline (PBS), then fixed with 100% ethanol for 30 min. Cells were incubated with rabbit antiserum (against PDCoV N protein) at 1:200 dilution and the reaction was kept at a 37°C incubation for 1 h followed by 6 times of washing with PBS. Then a secondary antibody of FITC-conjugated goat anti-rabbit antiserum (Solarbio, beijing, China) at 1:50 dilution was added and incubated at 37°C for 1 h. Cell wash was done in the same fashion as above. The IFA staining result was examined by using an fluorescent microscope.

### RNA isolation and quality control

Total RNA was extracted using a GenElute^™^ Total RNA Purification Kit (Sigma-Aldrich) following the manufacturer’s instructions. Polyacrylic acid amine gel electrophoresis was used to identify the RNA samples having no degradation or DNA pollution. Samples with OD (260/280) ratios in the range of 1.8–2.0 and OD (260/230) ratios from 1.8 to 2.2 met the requirement of sequencing as identified using a NanoPhotometer spectrophotometer. RNA samples with RNA integrity numbers (RINs) greater than 7 were selected for subsequent RNA sequencing which was performed using an Agilent 2100 bioanalyzer.

### qRT-PCR

Relative expression levels of 5 randomly selected genes were examined among the PK-15 cells at 0, 24, and 36 hpi by qRT-PCR using TB Green^TM^ Premix Ex Taq^TM^ II (Tli RNaseH Plus) (Takara Biomedical Technology). The relative gene expression levels of the 5 genes were normalized to *GAPDH* expression using the 2^-ΔΔCT^ method. The primers used in qRT-PCR were listed in S1 Table. The qRT-PCR reactions were performed on an ABI7500 StepOnePlus Real-Time PCR System (Thermo Fisher Scientific).

### cDNA library preparation and sequencing

The mRNAs with poly(A) in 3ʹUTR were enriched from the total RNA using Magnetic Beads Oligo(dT) (Dynal Biotech ASA), then disrupted into short fragments using a Magnesium RNA Fragmentation Module kit (NEB). With fragmented mRNAs as templates and random oligonucleotides as primers, the first strands of cDNA were synthesized. Subsequently, the second strands of cDNA were generated using DNA polymerase I and deoxyribonucleotide triphosphates (dNTPs). The terminals of purified double-strand cDNA sequences were repaired and provided with poly(A) and sequencing adapters. After ligating the adapters, the cDNA with 200 bp were screened using AMPure XP beads. Polymerase chain reaction (PCR) was used for cDNA amplification and carried out on a Veriti^™^ 96-well Thermal Cycler (Thermo Fisher Scientific). After further purifying the cDNA production using AMPure XP beads, a cDNA library was created. The insert sizes of cDNAs were detected using an Agilent 2100 bioanalyzer. High-quality cDNAs with insert sizes ranging from 250 to 300 bp were selected for further quantification via qRT-PCR. According to the requirements of deep sequencing, cDNAs having target concentrations and data sizes were pooled, followed by RNA sequencing, which was performed using an Illumina HiSeqTM 4000 platform (Novogene Corp.).

### Clean reads filtering and quality control

High-quality clean reads were screened from raw reads by removing reads with sequencing adapters or unknown nucleobases as well as low-quality reads in which the number of nucleobases with *Q*_Phred_ quality scores ≤ 20 were > 50% of the total read length. The distribution sequencing error rate was used to identify the quality of the data from Illumina sequencing. Q20 and Q30 represented the percent of nucleobases having 1% and 0.1% error rates, respectively, of the global nucleobase numbers determined using the Illumina sequencing platform.

### Genome mapping

The swine reference genome (NCBI genome database: *Sus scrofa* 11.1) was used as the reference genome for our RNA sequencing data. Genome mapping was performed using HISAT software (http://www.ccb.jhu.edu/software/hisat/index.shtml). The instructions of the HISAT software website was followed to prepare the data to be uploaded and also for the running analysis protocol.

### Differential expressed genes screening

The DESeq2 software, available from the link at https://bioconductor.org/packages/release/bioc/vignettes/DESeq2/inst/doc/DESeq2.html, was used for analysis of the differential expression levels of genes in each comparison group based on Anders and Huber’s report [31]. The thresholds of the *p*-values were determined using the false discovery rate (FDR, also referred to as *p*_adj_). The threshold for differentially expressed gene screening was established to be a value of |log2(fold change in a comparison group)| > 1 and a false discovery rate (FDR, or *p*_adj_) < 0.05. The FPKM (expected number of Fragments Per Kilobase of transcript sequence per Million base pairs sequenced) of each gene was calculated and used to represent the gene expression value [32].

### GO function and KEGG pathway enrichment analysis

Function classification and enrichment analysis of DEGs were performed using GOseq software based on GO (http://geneontology.org/page/go-enrichment-analysis). Pathway enrichment was analyzed based on the KEGG database (https://www.kegg.jp/kegg/kegg1.html) and carried out using the KOBAS 3.0 web server. GO terms or KEGG pathways with *p*-values < 0.05 were considered to be significantly enriched.

### Host cell endogenous miRNA prediction

Host cell endogenous miRNAs were predicted using RNAhybrid (https://bibiserv.cebitec.uni-bielefeld.de/rnahybrid/), PITA (http://genie.weizmann.ac.il/pubs/mir07/mir07_prediction.html), and miRanda (http://www.microrna.org/microrna/home.do). The miRNAs that had reliable binding sites to target genes and commonly existed in 3 prediction results were considered to be the contributors to the alteration of innate immune response induced by PDCoV. The binding sites between miRNA and target genes that had minimum free energy (MFE) levels < −10 kcal/mol and *p*-values < 0.05 as determined by RNAhybrid were considered to be reliable binding structures.

### Statistical analysis

The Student’s *t* test was used for statistical analyses in this study and carried out via SPSS Statistics 24.0.0 (IBM, USA), as described in a previous study [33]. Data were presented as the mean ± standard deviation (SD) of 2 independent experiments (*n* = 2). Data with *p* < 0.05 were deemed to be statistically significant.

## Results

### Identification of cytopathic effect and PDCoV N protein in PDCoV-inoculated PK-15 cells

**Fig 1:**
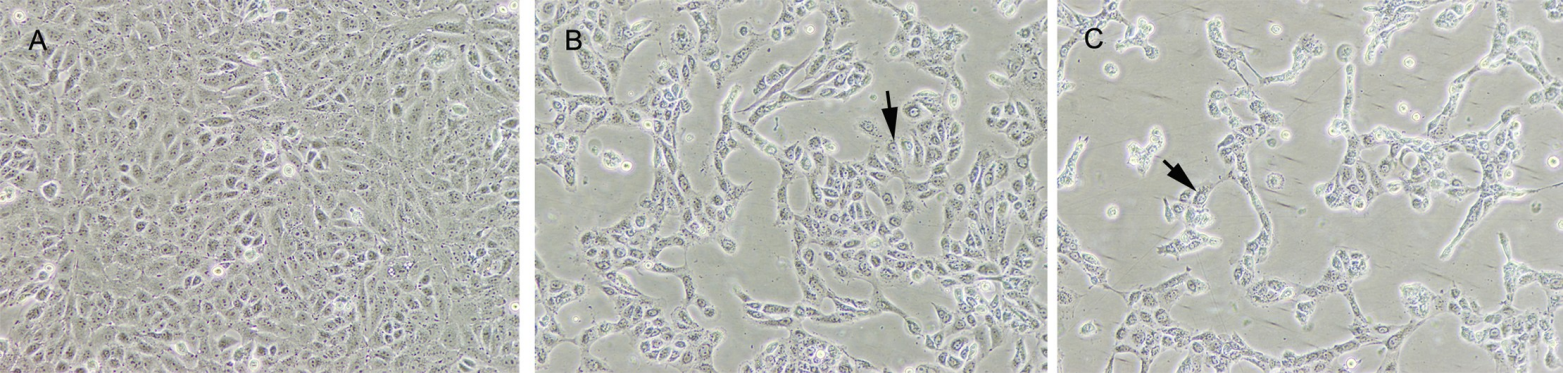
Cytopathic effect of PK-15 cell infections by PDCoV at 0, 24 and 36 hpi. In comparison with normal PK-15 cells (A), PDCoV infected cells at 24 hpi (B) exhibited CPE of rounded cells (arrow), shrinkaged cells and released cells from monolyer; after prolonged to 36 hpi, PDCoV infected showed more progressive development of CPEs (C).

**Fig 2:**
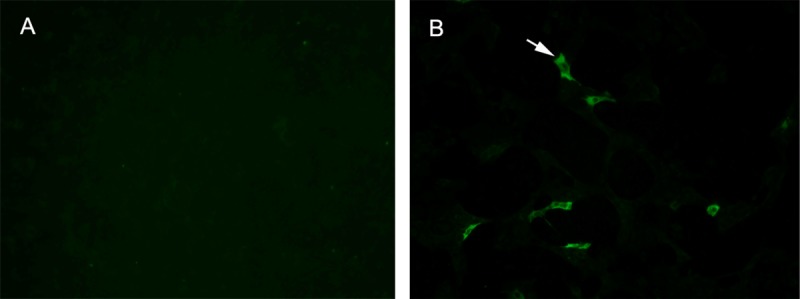
IFA staining on PK-15 cells. IFA stained positive (green fluorescence) on PDCoV-infected PK-15 cells (A) and negative on normal PK-15 cells (B).

PK-15 cells inoculated with PDCoV at 0.1 MOI were subsequently developed cytopathic effects (CPE) within 36 hpi. PDCoV induced CPEs on PK-15 cells was aggressively increased along with the increasing of PDCoV infection time (Fig 1). PDCoV-infected PK-15 cells at 24 hpi and 36 hpi yielded a same virus titer of 10^4.5^ TCID_50_/mL. The IFA stained positive to PDCoV tested on these CPE cells (Fig 2).

### Overview of RNA sequencing results

In order to further explore the alteration in the transcriptome profile of PK-15 cells during PDCoV infection, the global gene expression profiles of PK-15 cells after infection, including 24 hpi (INF_24h) and 36 hpi (INF_36h), as well as uninfected PK-15 cells (NC), which acted as negative controls, were obtained by RNA sequencing. A total of approximately 1.6 billion raw reads were initially generated through sequencing. After filtering out the reads with adapters, the reads with ambiguous nucleotides, and the reads of low quality, a total of nearly 156.2 million clean reads remained, with approximately 7.8 G bases per sample. Clean data were indicated by a low sequencing error rate (0.03%) and the high quality of identified nucleobases via sequencing (Q20 > 97%, Q30 > 92%). The guanine-cytosine (GC)-content of clean reads ranged from 52.94% to 54.2%. These high-quality clean reads were subsequently mapped to a reference genome (NCBI genome database: *Sus scrofa* 11.1) which exhibited total mapping rates > 94% and unique mapping rates > 91%. From this, approximately 21,416 expressed genes, including 5,874 novel genes, were identified per sample. The RNA sequencing statistics are listed in Table 1.

**Table 1:** Statistics of RNA sequencing results.

Sample	Raw reads	Clean reads	Clean bases (G)	Sequencing error rate (%)	Q20 (%)	Q30 (%)	GC content (%)	Total mapping rate (%)	Unique mapping rate (%)	Expressed genes	Novel genes
NC_1	26,873,398	26,397,492	7.92	0.03	97.18	92.50	53.06	95.18	92.56	21516	5591
NC_2	24,489,789	23,998,778	7.20	0.03	97.19	92.54	52.94	94.80	92.25	20813	5386
INF_24h1	29,972,480	29,121,085	8.74	0.03	97.46	93.44	53.61	94.10	91.20	22169	6160
INF_24h2	25,687,216	24,943,590	7.48	0.03	97.58	93.73	53.85	94.39	91.53	21425	5998
INF_36h1	29,874,857	28,998,514	8.70	0.03	97.60	93.72	54.20	94.32	91.54	21349	6097
INF_36h2	23,435,580	22,780,078	6.83	0.03	97.16	92.53	53.56	94.50	91.71	21224	6012

### Screening of differentially expressed genes

**Fig 3:**
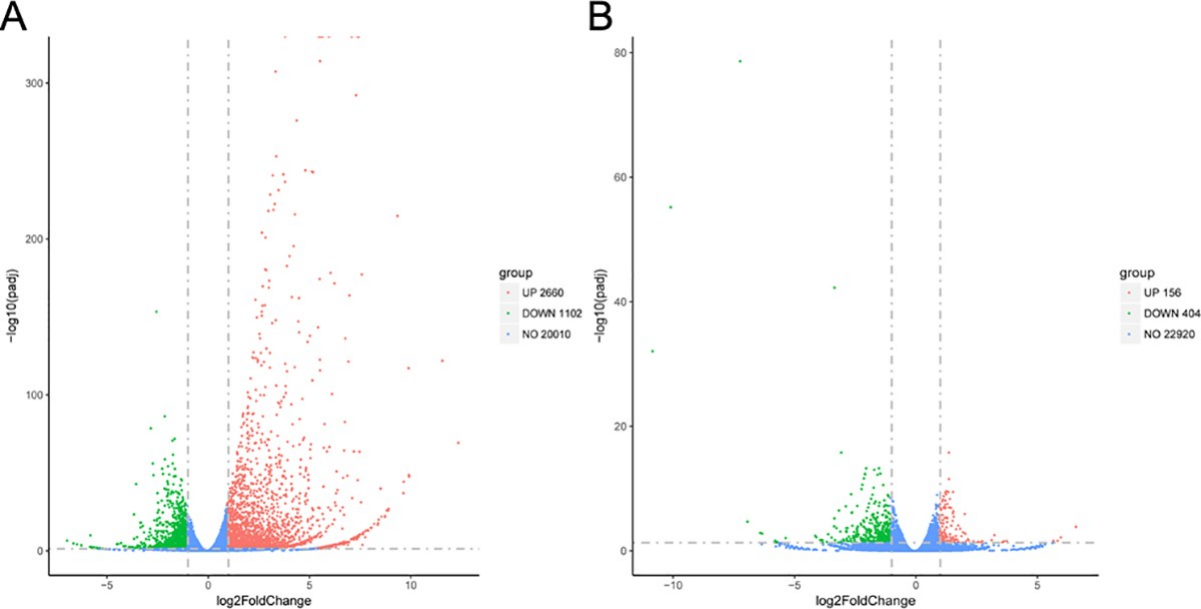
DEGs in INF_24 h versus NC and INF_36 h versus INF_24 h. (A): DEGs upregulated and downregulated on PK-15 cells at 24 hpi represented by red dots, uninfected PK-15 cells represented by green dots. Genes with no significant differences in expression represented in blue. (B): DEGs upregulated in INF_36 h compared to INF_24 h, the same color coded dots as (A).

The expression of all identified genes obtained through RNA sequencing were respectively compared for the group of uninfected PK-15 cells and cells at 24 hpi (INF_24h versus NC) as well as the group of PK-15 cells at 24 and 36 hpi (INF_36h versus INF_24h). Genes which differentially expressed in each comparison group with |log2(fold change in a comparison group)| > 1 and false discovery rate (FDR, or *p*_adj_) < 0.05 were considered to be differentially expressed genes (DEGs). For INF_24h versus NC, there were 3,762 DEGs, with 2,660 genes upregulated and 1,102 genes downregulated in INF_24h (Fig 3A; S2 Table). For INF_36h versus INF_24h, 560 DEGs were screened. Compared to INF_24h, 156 and 404 genes were respectively upregulated and downregulated in INF_36h (Fig 3B; S3 Table). The DEGs in these 2 comparisons were the genes that were mainly affected during PDCoV infection.

**Fig 4:**
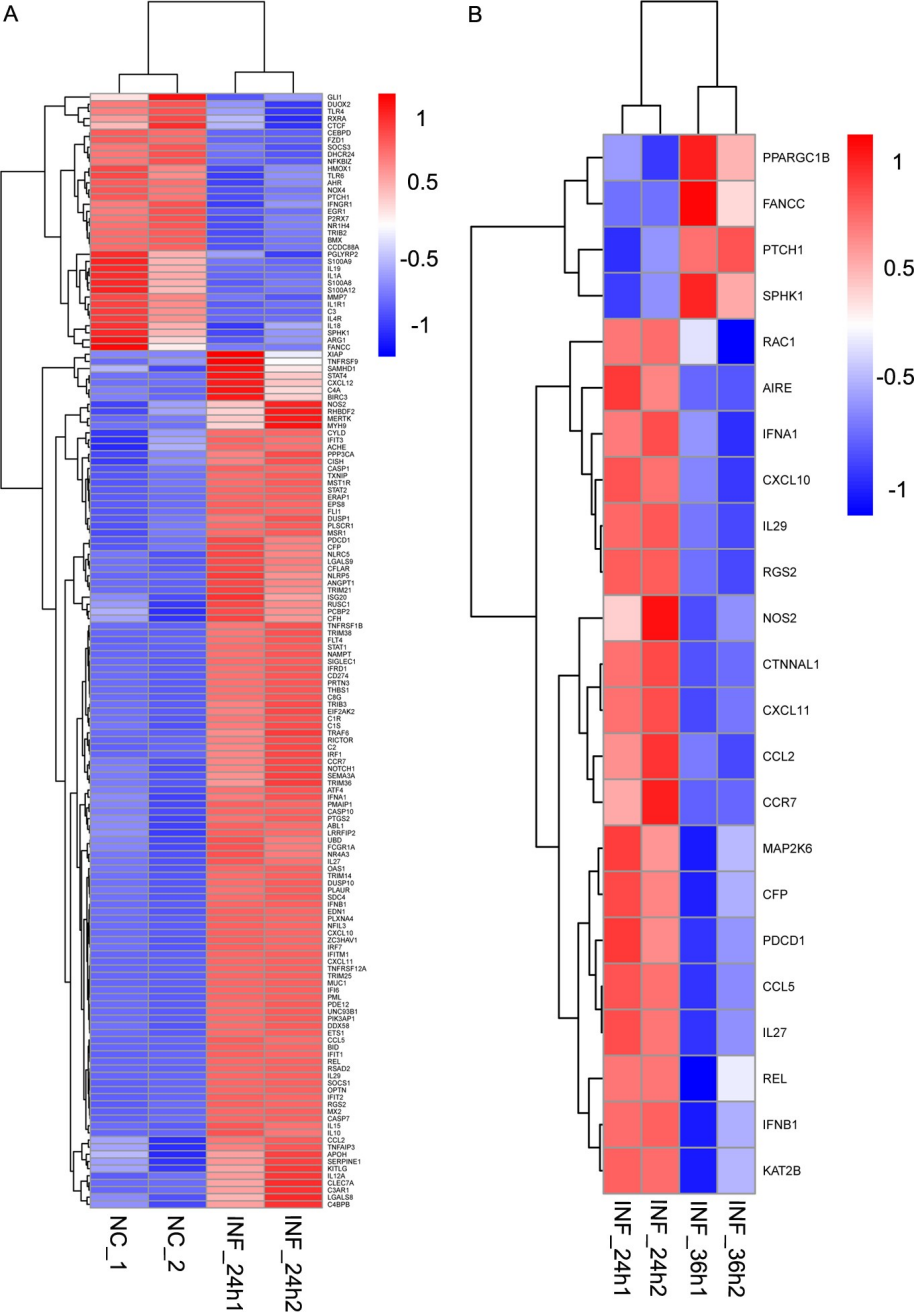
Innate immune-associated DEG. Innate immune-associated genes of DEGs in INF_24h versus NC (A) and INF_36h versus INF_24h (B) were illustrated in heatmaps that were created based on the expression of each DEG. (A) Genes with upregulated tendencies in INF_24h comparing to NC were displayed in red while genes with downregulated tendencies in INF_24h comparing to NC were shown in blue. (B) Genes with upregulated tendencies in INF_36h comparing to INF_24h were represented in red while genes with downregulated tendencies were shown in blue.

In order to identify the alteration of innate immune-related genes in host cells as PDCoV post infection time increased, porcine innate immune genes were further screened. Up to date, porcine innate immune genes have not been collected into a database which can be conveniently updated frequently. In addition, the anatomy, organization, and response of pig immune system are very similar to humans’ [34]. Thus, the porcine innate immune genes that included in the DEGs were screened by referring to the innate immune genes list of the InnateDB (http://www.innatedb.ca/redirect.do?go=aboutIDB) which provides a total of 1,040 curated human genes related to the innate immune response. There were 156 and 23 swine innate immune genes that were differentially expressed in INF_24h versus NC and INF_36h versus INF_24h, respectively. In INF_24h versus NC, innate immune DEGs with 120 upregulated and 36 downregulated tendencies were identified in INF_24h compared to NC (Fig 4A). In INF_36h versus INF_24h, 19 genes were downregulated in INF_36h while 4 genes were upregulated (Fig 4B). Of note, most of the differential expressed innate immune genes including *I-IFNs* such as *IFN-α1* and *IFN-β1* (also known as *IFN-β*) were upregulated in cells at 24 hpi comparing to uninfected cells, and downregulated in cells at 36 hpi comparing to cells at 24 hpi, which indicated that innate immune response was initially enhanced and then inhibited in cells at 24 hpi and 36 hpi, respectively.

**Fig 5:**
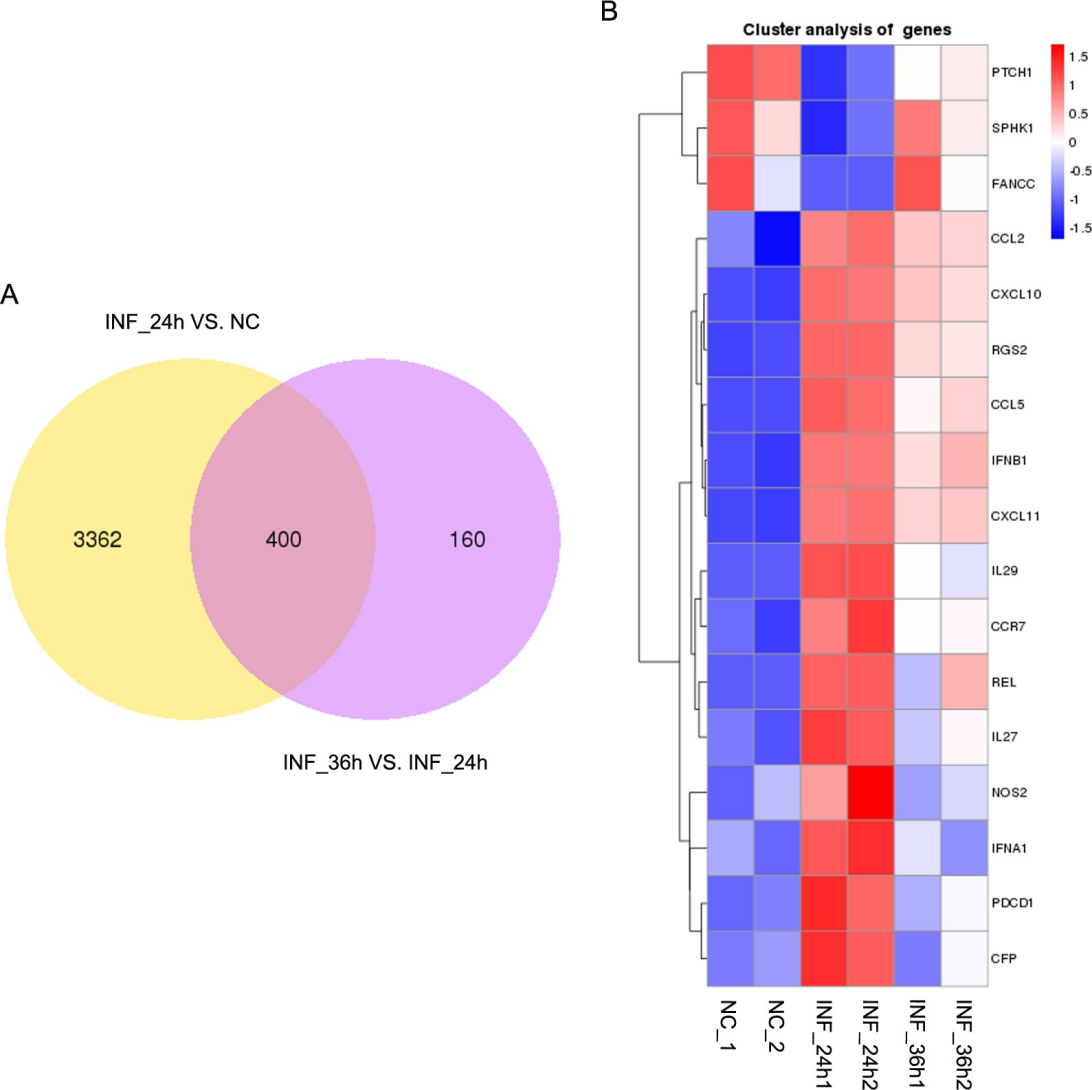
Common genes belonging to DEGs in INF_24h versus NC and INF_36h versus INF_24h. (A) A total of 400 DEGs were commonly expressed in the 2 comparison groups. (B) Innate immune-associated genes of DEGs commonly shared in cells at 0, 24, and 36 hpi were illustrated in a heatmap. The heatmap was constructed based on the expression for each DEG with 2 replicates examined.

**Fig 6:**
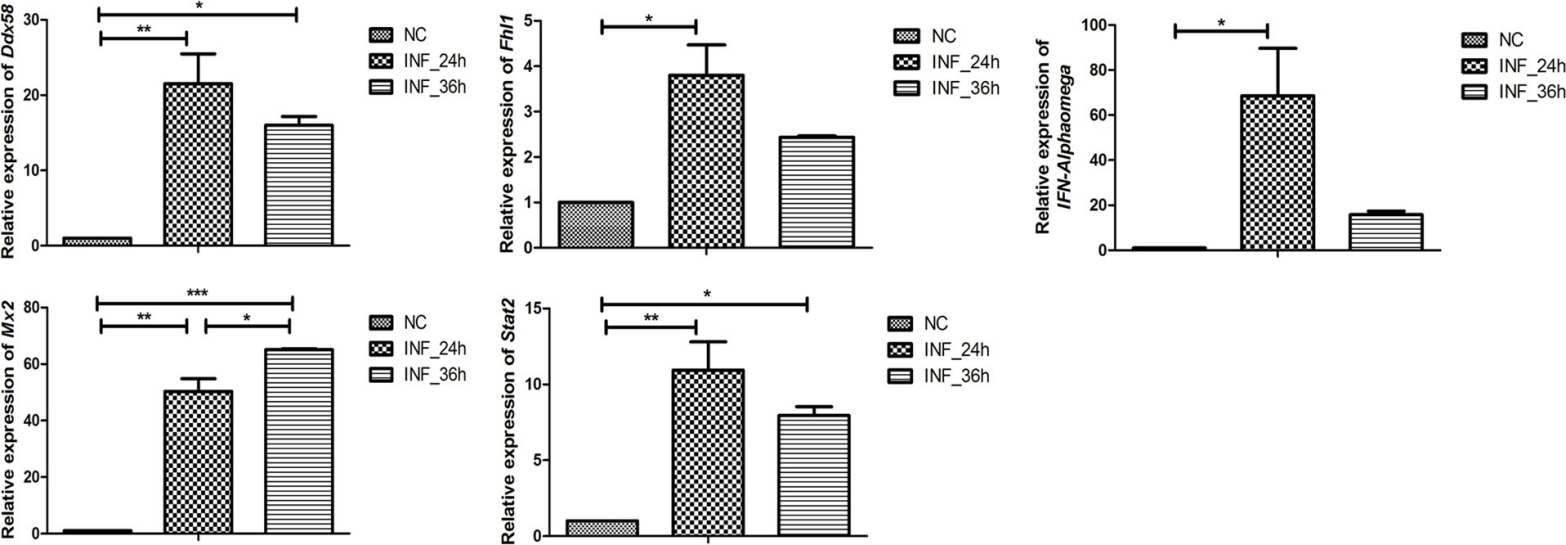
Relative expression of 5 randomly selected genes among PK-15 cells at 0, 24 and 36 hpi. Relative expression of all of the genes were coincident with our RNA sequencing results. Data are displayed as mean ± SD (*n* = 2); *p < 0.05; **p < 0.01; ***p < 0.001.

In addition, these 2 comparison groups shared 400 DEGs (Fig 5A). A total of 17 DEGs including *IFN-α1* and *IFN-β1* belonged to porcine innate immune-associated genes (Fig 5B). Five of the 17 genes were randomly selected for validation via qRT-PCR, and the results showed that the relative expressions of these genes were coincident with our RNA sequencing results (Fig 6).

### GO function enrichment analysis

**Fig 7:**
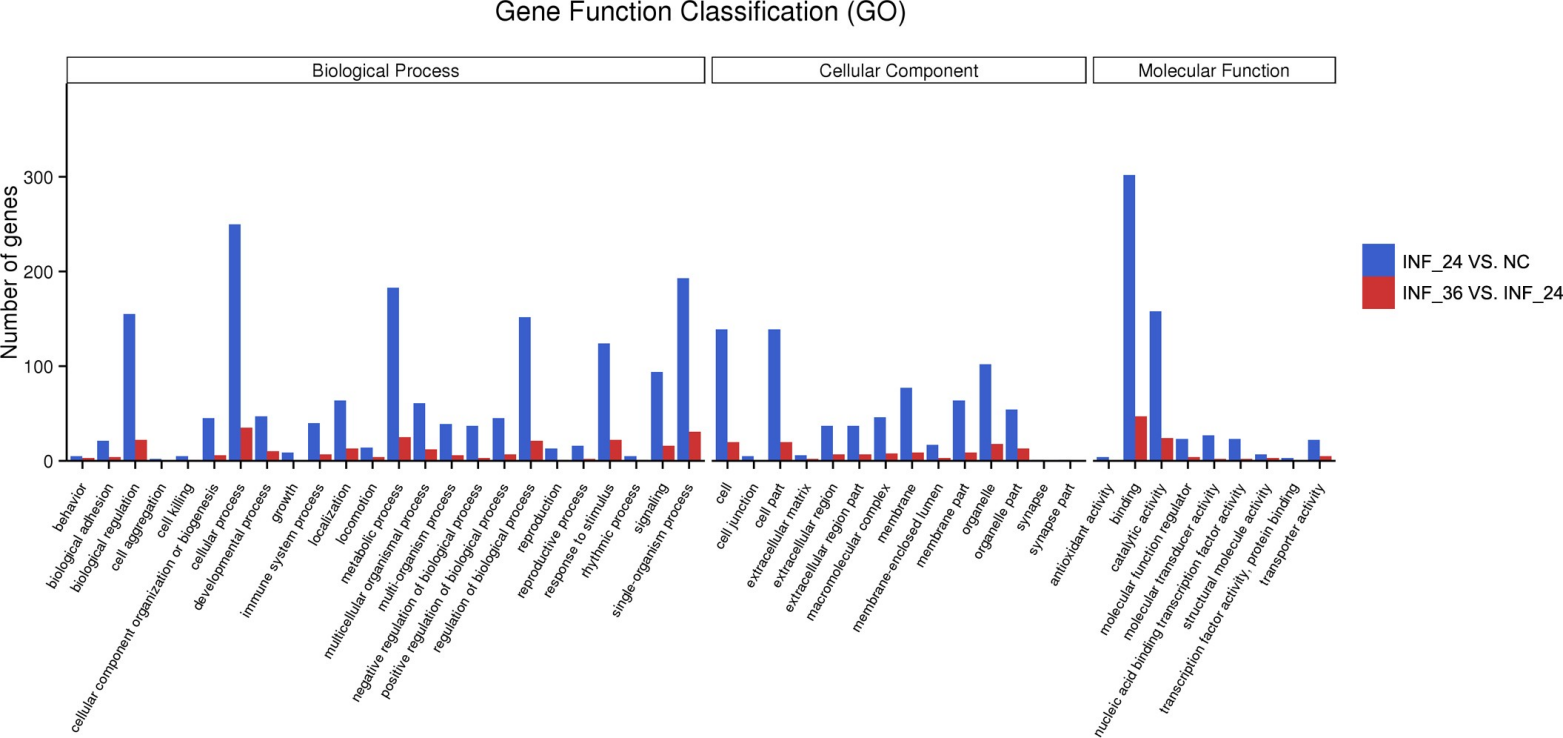
Gene function classifications of DEGs in 2 comparison groups. Gene function classifications of DEGs in INF_24h versus NC and INF_36h versus INF_24h comparisons are represented by blue and red bars, respectively.

**Fig 8:**
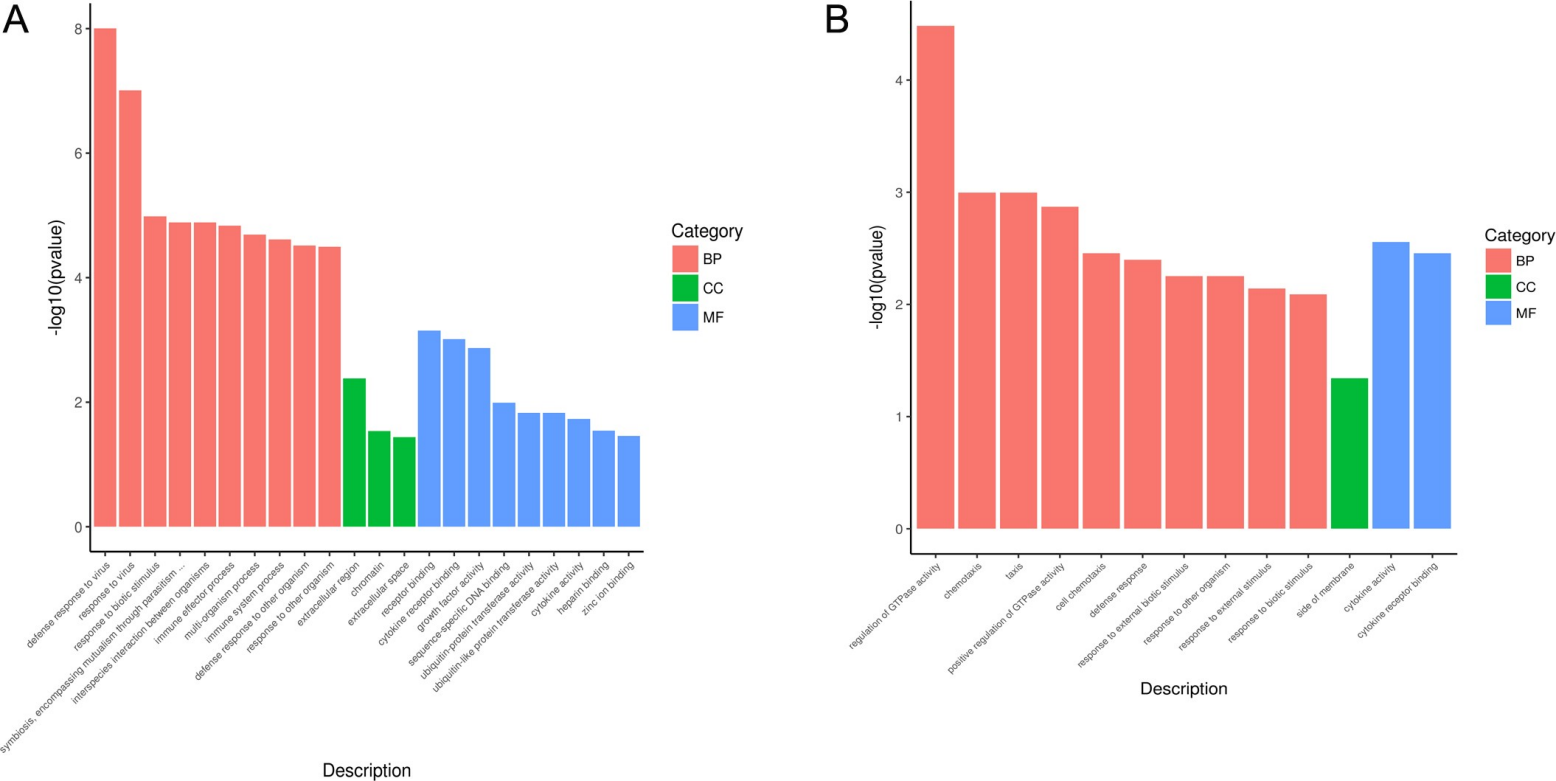
GO function enrichment results of DEGs in 2 comparison groups. Top 10 significantly enriched GO terms of each category, including biological process (BP), cellular component (CC), and molecular function (MF) were displayed in this figure (A: INF_24h versus NC; B: INF_36h versus INF_24h). Enriched GO terms with *p* < 0.05 were considered to be statistically significant.

The DEGs in the 2 comparison groups were annotated by the Gene Ontology (GO) function database, which divided them into 3 macroscopic groups, including biological process (BP), cellular component (CC), and molecular function (MF). The number of DEGs in the INF_24h versus NC comparison that fell into each Gene function classification were higher than the number of DEGs in the INF_36h versus INF_24h comparison (Fig 7). Also, in relation to INF_36h versus INF_24h, the DEGs annotated by GO terms in INF_24h versus NC were more abundant (Fig 8).

To explore the function of genes that affected during the alteration of innate immune response caused by PDCoV, DEGs whose relative expression tendencies were consistent with most of the differential expressed host innate immune genes screened in the present study were further analyzed using GO function enrichment. Namely, DEGs upregulated in cells at 24 hpi compared to uninfected cells and DEGs downregulated in cells at 36 hpi compared to cells at 24 hpi were selected for further analysis with GO function enrichment. For INF_24h versus NC, genes which were upregulated in cells at 24 hpi were enriched in 99 terms of BP, 3 terms of CC, and 9 terms of MF (S4 Table). Most of these genes were associated with response against viruses, host defense response, and immune response (Fig 8A). In another comparison group, genes with downregulated tendencies in cells at 36 hpi were enriched in 65 terms of BP, 1 term of CC, and 2 terms of MF (S5 Table). These genes mainly enriched in the functions related to host defense responses, such as defense response, response to external biotic stimuli, response to external stimuli, and in the functions of cytokines involved in immune and innate immune response, such as cytokine activity and cytokine receptor binding (Fig 8B). Since genes annotated with GO terms associated with immune and innate immune responses were commonly shared, the enriched immune and innate immune response-related GO terms were both selected to represented (Tables 2 and 3).

**Table 2:** GO function enrichment results of upregulated DEGs in cells at 24 hpi comparing to unfected cells.

Category	ID	Description	*P-*value	Gene name	Count
BP	GO:0002252	Immune effector process	1.47E-05	*RSAD2*, *OAS1*, *DDX58*, *MX2*, *STAT1*, *STAT2*, *C1S*, *IL27*, *IFNA1*, *IRF1*, *TRAF6*, *ISG20*, *IL10*, *AZU1*	15
BP	GO:0002376	Immune system process	2.42E-05	*RSAD2*, *OAS1*, *DDX58*, *MX2*, *STAT1*, *STAT2*, *OLR1*, *PSMB8*, *SOX9*, *CCL5*, *SDC4*, *C1S*, *CCL2*, *IL15*, *IL27*, *IL12A*, *IFNA1*, *IRF1*, *TRAF6*, *ISG20*, *IL10*, *AZU1*, *ESR1*	31
BP	GO:0006955	Immune response	8.04E-05	*RSAD2*, *OAS1*, *DDX58*, *MX2*, *STAT1*, *STAT2*, *CCL5*, *C1S*, *CCL2*, *IL15*, *IL27*, *IL12A*, *IRF1*, *TRAF6*, *ISG20*, *IL10*, *AZU1*, *ESR1*	25
BP	GO:0001819	Positive regulation of cytokine production	1.45E-03	*RSAD2*, *DDX58*, *STAT1*, *IL27*, *IRF1*, *TRAF6*, *IL10*, *AZU1*	8
BP	GO:0045087	Innate immune response	1.70E-03	*RSAD2*, *OAS1*, *DDX58*, *MX2*, *STAT1*, *STAT2*, *CCL5*, *C1S*, *CCL2*, *IL27*, *IRF1*, *ISG20*, *ESR1*	13
BP	GO:0032635	Interleukin-6 production	2.99E-03	*DDX58*, *GHSR*, *TRAF6*, *NOS2*, *IL10*	5
BP	GO:0001817	Regulation of cytokine production	4.50E-03	*RSAD2*, *DDX58*, *STAT1*, *IL27*, *GHSR*, *IRF1*, *TRAF6*, *NOS2*, *IL10*, *AZU1*	10
BP	GO:0042035	Regulation of cytokine biosynthetic process	4.97E-03	*IL27*, *GHSR*, *IRF1*, *TRAF6*, *AZU1*	5
BP	GO:0042089	Cytokine biosynthetic process	7.74E-03	*IL27*, *GHSR*, *IRF1*, *TRAF6*, *AZU1*	5
BP	GO:0042107	Cytokine metabolic process	7.74E-03	*IL27*, *GHSR*, *IRF1*, *TRAF6*, *AZU1*	5
BP	GO:0001816	Cytokine production	1.24E-02	*RSAD2*, *DDX58*, *STAT1*, *IL27*, *GHSR*, *IRF1*, *TRAF6*, *NOS2*, *IL10*, *AZU1*	10
BP	GO:0002758	Innate immune response-activating signal transduction	2.05E-02	*RSAD2*, *DDX58*, *IRF1*, *ESR1*	4
BP	GO:0002218	Activation of innate immune response	2.93E-02	*RSAD2*, *DDX58*, *IRF1*, *ESR1*	4
BP	GO:0007249	I-kappaB kinase/NF-kappaB signaling	2.93E-02	*STAT1*, *OPTN*, *TRAF6*, *ESR1*	4
BP	GO:0045089	Positive regulation of innate immune response	2.93E-02	*RSAD2*, *DDX58*, *IRF1*, *ESR1*	4
BP	GO:0002682	Regulation of immune system process	2.99E-02	*RSAD2*, *DDX58*, *STAT1*, *SOX9*, *CCL5*, *SDC4*, *C1S*, *CCL2*, *IL27*, *IRF1*, *TRAF6*, *IL10*, *ESR1*	13
BP	GO:0002757	Immune response-activating signal transduction	3.74E-02	*RSAD2*, *DDX58*, *IRF1*, *TRAF6*, *ESR1*	5
BP	GO:0002697	Regulation of immune effector process	4.71E-02	*RSAD2*, *DDX58*, *IL27*, *TRAF6*, *IL10*	5
MF	GO:0005126	Cytokine receptor binding	9.74E-04	*CCL5*, *CCL2*, *IL15*, *IL27*, *BDNF*, *IL12A*, *IFNA1*, *KITLG*, *TRAF6*	13
MF	GO:0005125	Cytokine activity	1.86E-02	*NAMPT*, *CCL5*, *CCL2*, *IL15*, *IL27*, *IL12A*, *IFNA1*, *IL10*	10

Immune and innate immune response-related GO terms were represented in this table.

**Table 3:** GO function enrichment results of downregulated DEGs in cells at 36 hpi comparing to cells at 24 hpi.

Category	ID	Description	*P-*value	Gene name	Count
BP	GO:0002376	Immune system process	1.69E-02	*CCL2*, *CCL5*, *IFNA1*, *AZU1*, *IL27*	7
BP	GO:0006955	Immune response	1.87E-02	*CCL2*, *CCL5*, *AZU1*, *IL27*	6
BP	GO:0042035	Regulation of cytokine biosynthetic process	2.19E-02	*AZU1*, *IL27*	2
BP	GO:0042089	Cytokine biosynthetic process	2.59E-02	*AZU1*, *IL27*	2
BP	GO:0042107	Cytokine metabolic process	2.59E-02	*AZU1*, *IL27*	2
BP	GO:0001817	Regulation of cytokine production	3.74E-02	*AZU1*, *IL27*, *NOS2*	3
MF	GO:0005125	Cytokine activity	2.78E-03	*CCL2*, *CCL5*, *IFNA1*, *IL27*	5
MF	GO:0005126	Cytokine receptor binding	3.50E-03	*CCL2*, *CCL5*, *IFNA1*, *IL27*	5

Immune and innate immune response-related GO terms were represented in this table.

### KEGG pathway enrichment analysis

**Fig 9:**
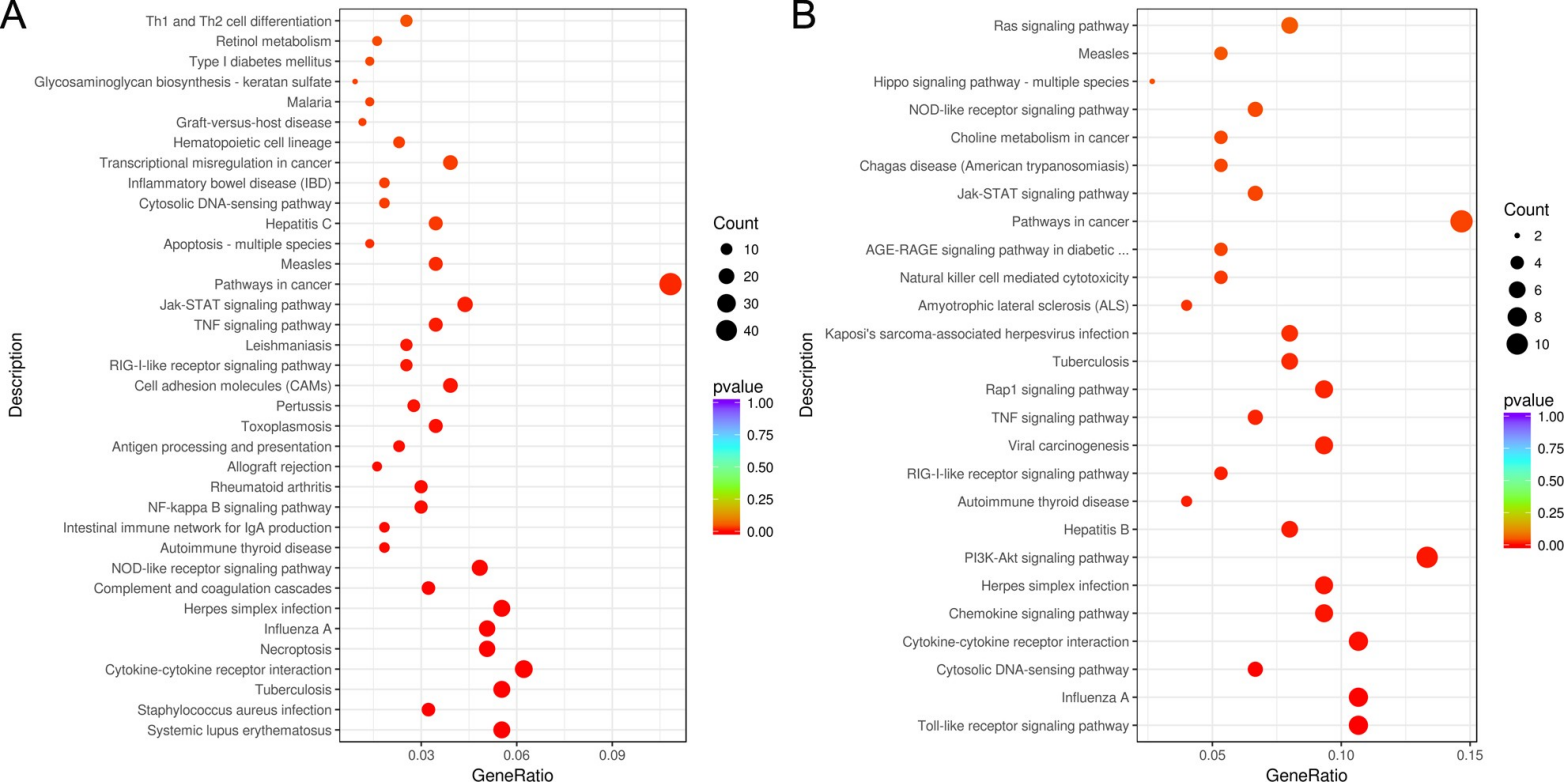
KEGG pathway enrichment results of DEGs in 2 comparison groups. KEGG signaling pathways which were significantly enriched in INF_24h versus NC and INF_36h versus INF_24h are represented in (A) and (B), respectively. Enriched signaling pathways with *p* < 0.05 were considered to be statistically significant.

DEGs used in GO function enrichment analysis were also selected for analysis in Kyoto Encyclopedia of Genes and Genomes (KEGG) pathway enrichment. A total of 36 signaling pathways (Fig 9A), including 6 innate immune response-associated pathways (Table 4), were enriched in INF_24h versus NC. Additionally, a total of 26 signaling pathways (Fig 9B), including 9 related to innate immune response (Table 5), were enriched in INF_36h versus INF_24h. This finding indicates that extensive bioprocesses were affected by PDCoV infection. Of note, 5 signaling pathways were commonly represented in the 2 enrichment results. These innate immune-associated pathways were mainly affected during PDCoV enhanced or inhibited innate immune response of PK-15 cells. In addition, 3 of the 5 pathways belong to pattern recognition receptors (PRRs) mediated signalings, including NOD-like receptor signaling pathway, RIG-I-like receptor signaling pathway, and cytosolic DNA-sensing pathway, which can mediate the *I-IFN* production. One of the 5 pathways, Jak/STAT signaling pathway, belongs to the downstream signaling of *I-IFN* response which can mediate the production of ISGs. These 4 pathways are known to play important roles in the process that *I-IFN* develops antiviral function in innate immune response.

**Table 4:** KEGG pathway enrichment results of upregulated DEGs in INF_24h comparing to NC.

ID	Description	Gene ratio	*P*-value	Gene name	Count
ssc04060	Cytokine-cytokine receptor interaction	27/434	3.46E-04	*IFNB1*, *CXCL10*, *IFN*-*ALPHAOMEGA*, *TNFRSF12A*, *CXCL11*, *CCL5*, *TNFSF13B*, *TNFRSF1B*, *CCL2*, *IL15*, *CCR7*, *FLT4*, *IL22RA1*, *IL12A*, *IFNA1*, *KITLG*, *IL10RA*, *IL10*, *IL12RB1*, *AMHR2*, *CXCL12*, *TNFRSF9*, *IL11*, *HGF*, *MPL*, *AMH*, *TNFRSF11A*	27
ssc04621	NOD-like receptor signaling pathway	21/434	1.01E-03	*GBP1*, *OAS1*, *IFNB1*, *STAT1*, *STAT2*, *IFN*-*ALPHAOMEGA*, *LOC100155195*, *TXNIP*, *CASP1*, *NAMPT*, *LOC100156073*, *IRF7*, *CCL5*, *LOC100523492*, *CCL2*, *TNFAIP3*, *IFNA1*, *TRAF6*, *BIRC3*, *ERBIN*, *XIAP*	21
ssc04064	NF-kappa B signaling pathway	13/434	1.76E-03	*DDX58*, *TRIM25*, *PTGS2*, *TNFSF13B*, *VCAM1*, *CARD11*, *TNFAIP3*, *CFLAR*, *TRAF6*, *BIRC3*, *CXCL12*, *XIAP*, *TNFRSF11A*	13
ssc04622	RIG-I-like receptor signaling pathway	11/434	4.75E-03	*IFNB1*, *DDX58*, *CXCL10*, *IFN*-*ALPHAOMEGA*, *TRIM25*, *CASP10*, *IRF7*, *CYLD*, *IL12A*, *IFNA1*, *TRAF6*	11
ssc04630	Jak-STAT signaling pathway	19/434	6.74E-03	*IFNB1*, *STAT1*, *STAT2*, *IFN*-*ALPHAOMEGA*, *SOCS1*, *FHL1*, *IL29*, *PIM1*, *IL15*, *IL22RA1*, *IL12A*, *IFNA1*, *CISH*, *IL10RA*, *IL10*, *IL12RB1*, *STAT4*, *IL11*, MPL	19
ssc04623	Cytosolic DNA-sensing pathway	8/434	2.43E-02	*IFNB1*, *DDX58*, *CXCL10*, *IFN*-*ALPHAOMEGA*, *CASP1*, *IRF7*, *CCL5*, *IFNA1*	8

Innate immune response-related pathways were represented in this table.

**Table 5:** KEGG pathway enrichment results of downregulated DEGs in INF_36h comparing to INF_24h.

ID	Description	Gene ratio	*P-*value	Gene name	Count
ssc04620	Toll-like receptor signaling pathway	8/75	1.77E-05	*CXCL10*, *IFN*-*ALPHAOMEGA*, *IFNB1*, *CCL5*, *CXCL11*, *IFNA1*, *RAC1*, *MAP2K6*	8
ssc04623	Cytosolic DNA-sensing pathway	5/75	3.28E-04	*CXCL10*, *IFN*-*ALPHAOMEGA*, *IFNB1*, *CCL5*, *IFNA1*	5
ssc04060	Cytokine-cytokine receptor interaction	8/75	2.53E-03	*CXCL10*, *IFN*-*ALPHAOMEGA*, *IFNB1*, *CCL2*, *CCL5*, *CXCL11*, *IFNA1*, *CCR7*	8
ssc04151	PI3K-Akt signaling pathway	10/75	4.74E-03	*IFN*-*ALPHAOMEGA*, *IFNB1*, *YWHAZ*, *IFNA1*, *RAC1*, *FGF18*, *GNG11*, *PDGFC*, *COL9A2*, *COL4A6*	10
ssc04622	RIG-I-like receptor signaling pathway	4/75	7.52E-03	*CXCL10*, *IFN*-*ALPHAOMEGA*, *IFNB1*, *IFNA1*	4
ssc04015	Rap1 signaling pathway	7/75	9.37E-03	*RALGDS*, *CTNND1*, *SIPA1L2*, *RAC1*, *FGF18*, *MAP2K6*, *PDGFC*	7
ssc04630	Jak-STAT signaling pathway	5/75	3.14E-02	*IFN*-*ALPHAOMEGA*, *IFNB1*, *IL29*, *FHL1*, *IFNA1*	5
ssc04621	NOD-like receptor signaling pathway	5/75	3.35E-02	*IFN*-*ALPHAOMEGA*, *IFNB1*, *CCL2*, *CCL5*, *IFNA1*	5
ssc04014	Ras signaling pathway	6/75	4.82E-02	*RALGDS*, *RAC1*, *REL*, *FGF18*, *GNG11*, *PDGFC*	6

Innate immune response-related pathways were represented in this table.

### Host cell endogenous miRNA prediction

**Fig 10:**
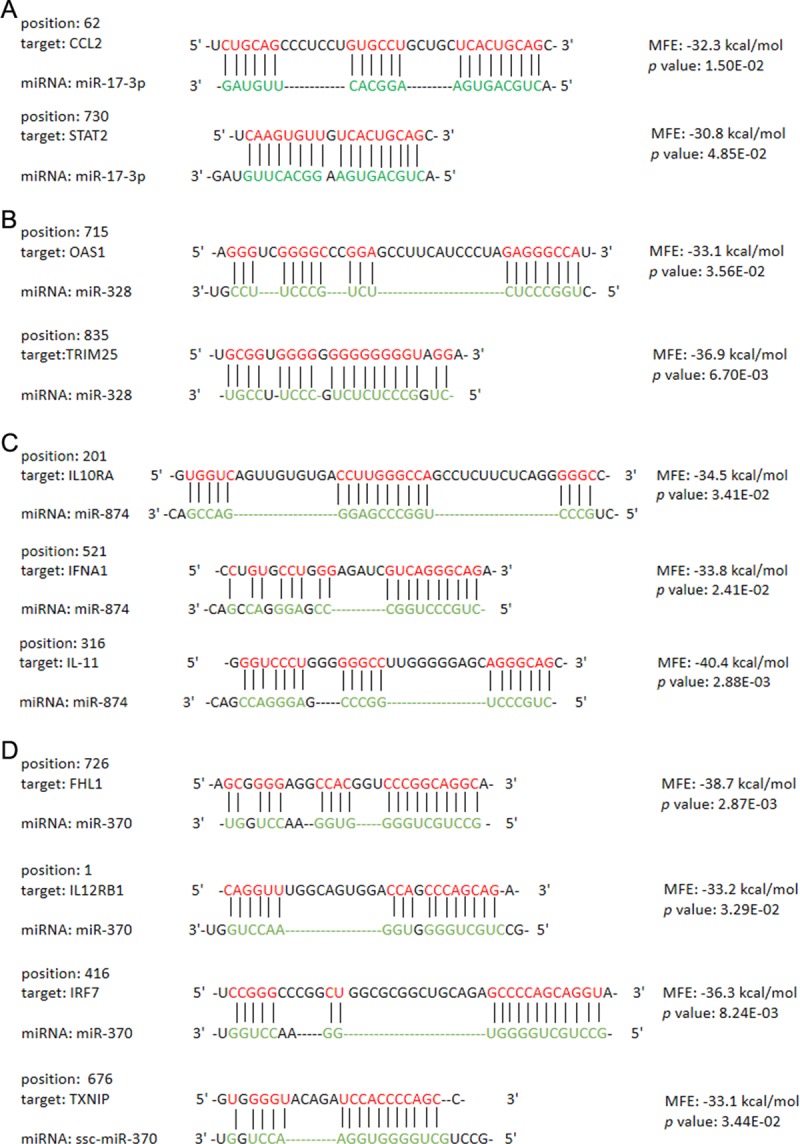
Binding sites between miRNAs and their target genes covered in innate immune signaling pathways. Four miRNAs with multiple targets were selected to display the binding sites (A: miR-17-3p; B: miR-328; C: miR-874; D: miR-370). The results of binding sites were predicted by RNAhybrid. The binding sites with a minimum free energy (MFE) < −10 kcal/mol and a *p*-value < 0.05 were considered to be stable binding site structures.

It is well known that host cell endogenous miRNAs are crucial contributors in modulating host cells innate immune response which affected by coronaviruses. Given this, the present study further predicted PK-15 cell-derived miRNAs which may involved in the alteration of PK-15 cell innate immune response caused by PDCoV infection. Considering the importance of the 4 innate immune signaling pathways in the process that *I-IFN* developed antiviral function, DEGs which were covered in these signaling pathways were collected for miRNA prediction. A total of 16 miRNAs were likely corelated with DEGs, of which 12 could modulate 1 or more innate immune signaling pathway by directly regulating unique target genes (Table 6), and 4 could modulate 2 or more innate immune signaling pathways by directly regulating multiple target genes (Table 7; Fig 10).

**Table 6:** Predicted miRNAs with unique targets.

Species	MiRNA	Target gene	Innate immune signaling pathway
*Sus scrofa*	miR-195	*IFN*-*ALPHAOMEGA*	NOD-like receptor signaling pathway, RIG-I-like receptor signaling pathway, Jak-STAT signaling pathway, Cytosolic DNA-sensing pathway
*Sus scrofa*	miR-7137-3p	*OAS1*	NOD-like receptor signaling pathway
*Sus scrofa*	miR-4334-5p	*TNFAIP3*	NOD-like receptor signaling pathway
*Sus scrofa*	miR-139-3p	*TNFAIP3*	NOD-like receptor signaling pathway
*Sus scrofa*	miR-493-3p	*IFNA1*	NOD-like receptor signaling pathway, RIG-I-like receptor signaling pathway, Jak-STAT signaling pathway, Cytosolic DNA-sensing pathway
*Sus scrofa*	miR-9822-3p	*CASP1*	NOD-like receptor signaling pathway, Cytosolic DNA-sensing pathway
*Sus scrofa*	let-7a	*CISH*	Jak-STAT signaling pathway
*Sus scrofa*	miR-4335	*TNFAIP3*	NOD-like receptor signaling pathway
*Sus scrofa*	miR-1306-5p	*TRIM25*	RIG-I-like receptor signaling pathway
*Sus scrofa*	miR-9853-5p	*IL12RB1*	Jak-STAT signaling pathway
*Sus scrofa*	miR-92b-5p	*TNFAIP3*	NOD-like receptor signaling pathway
*Sus scrofa*	miR-671-5p	*CYLD*	RIG-I-like receptor signaling pathway

**Table 7:** Predicted miRNAs with multiple targets.

Species	MiRNA	Target gene	Innate immune signaling pathway
*Sus scrofa*	miR-17-3p	*CCL2*	NOD-like receptor signaling pathway
		*STAT2*	NOD-like receptor signaling pathway, Jak-STAT signaling pathway
*Sus scrofa*	miR-328	*OAS1*	NOD-like receptor signaling pathway
		*TRIM25*	RIG-I-like receptor signaling pathway
*Sus scrofa*	miR-874	*IL10RA*	Jak-STAT signaling pathway
		*IFNA1*	NOD-like receptor signaling pathway, RIG-I-like receptor signaling pathway, Jak-STAT signaling pathway, Cytosolic DNA-sensing pathway
		*IL11*	Jak-STAT signaling pathway
*Sus scrofa*	miR-370	*IL12RB1*	Jak-STAT signaling pathway
		*FHL1*	Jak-STAT signaling pathway
		*IRF7*	NOD-like receptor signaling pathway, RIG-I-like receptor signaling pathway, Cytosolic DNA-sensing pathway
		*TXNIP*	NOD-like receptor signaling pathway

## Discussion

TGEV, PEDV, and PDCoV are coronaviruses causing most severe swine enteric disease problems and resulting in significant economic losses in swine industry [12]. It has been reported that pigs experiencing severe diarrhea are frequently coinfected with TGEV, PEDV, and PDCoV. However, even in the absence of TGEV and PEDV, PDCoV has high pathogenicity, making it worthy of significant concern. In general, PDCoV was the only pathogenic factor among these three viruses, which could be detected in feces and intestinal samples and associated with outbreaks of swine diarrheal disease in the U.S. in 2014 [12]. To date, the infection mechanisms of TGEV and PEDV at the mRNA or miRNA levels have already been revealed by high-throughput sequencing, which has delineated the key genes (or miRNAs) and signaling pathways involved in the process of viral infection [26, 35]. However, analogous PDCoV research still lags behind. The present study is the first related to the discovery of the infection mechanism of PDCoV at the transcriptome level by RNA sequencing.

Escaping the host cell innate immune response is the key stage in the successful infection of PDCoV in host cells. To date, research studies have made great progress revealing the strategies that PDCoV utilizes to escape *I-IFN*-associated host cell innate response. Nevertheless, we still need further research studies to completely understand the complex molecular mechanism of the alteration of innate immune response affected by PDCoV. Given this, our study focused primarily on discovering innate immune-associated genes and signaling pathways that altered by PDCoV infection in PK-15 cells.

Reported studies indicated that multiple cell lines including ST, LLC-PK and IPI-2I were permissive to PDCoV [36,37]. We found the PK-15 cell line was permissive to PDCoV in this study. However, although the degree of CPE in PK-15 cells from 24 hpi to 36 hpi showed an increasing tendency, cells at 12 h different post infection (pi) time yielded a similar virus titer. The irrelevance between the number of infected cells and virus titer at each pi time point could result from susceptibility of PK-15 to PDCoV, which was different from other cell lines of permissive to PDCoV.

To further investigate the mechanism of PDCoV infection, all of the expressed genes were identified in PK-15 cells at 0, 24 and 36 hpi through RNA sequencing. By comparing the expressed genes in cells at 0 and 24 hpi, we found that most of the innate immune-associated genes were significantly upregulated at 24 hpi comparing to cells without PDCoV infection. The results indicated that *I-IFN*-mediated innate immune response was triggered during PDCoV at an early infection time point (24 hpi). In the comparison between cells at 24 hpi and 36 hpi, the PRRs and ISGs described above which were elevated significantly in INF_24h versus NC were not downregulated significantly in cells at 36 hpi comparing to cells at 24 hpi, but the significant inhibition of *I-IFNs* expression still can be found. The result indicated that the *I-IFNs* production was reduced at 36 hpi comparing to 24 hpi which also indicated that the *I-IFN* response might be inhibited at 36 hpi induced by PDCoV. The reason that the PRRs or ISGs described above were not significantly down-regulated at this infection time point may due to there are other key molecules which regulated by PDCoV for suppression of *I-IFNs* production or *I-IFN* response at 36 hpi. Besides, the results also indicated that the genes which regulated by PDCoV in a relative early infection time (24 hpi) and a relative late infection time (36 hpi) for innate immune response modulation were different.

The results of GO function and KEGG signaling pathway enrichment of differentially expressed genes possessing the expression tendency consistent with most of the innate immune-associated genes in INF_24h versus NC as well as INF_36h versus INF_24h revealed that extensive gene functions and signaling pathways including innate immune-related ones were affected at different infection time of PDCoV. Additionally, we found that the GO terms and KEGG signaling pathways which enriched in two comparison groups were distinct. These findings suggested that the gene functions and signaling pathways which affected by PDCoV for modulation of innate immune response at different post infection times were complicated and different.

Five innate immune-associated signaling pathways which were commonly enriched in 2 comparison groups are primarily affected during the alteration of innate immune response of host cells which caused by PDCoV infection. Of note, 4 of the 5 signaling pathways including NOD-like receptor signaling pathway, RIG-I-like receptor signaling pathway, Jak-STAT signaling pathway, and cytosolic DNA-sensing pathway play crucial roles in the process that *I-IFN* develops antiviral function in innate immune response. Moreover, NF-kappa B signaling pathway is specifically enriched in INF_24h versus NC, while Toll-like receptor signaling pathway, PI3K-Akt signaling pathway, Rap1 signaling pathway and Ras signaling pathway are specifically enriched in INF_36h versus INF_24h. Recent studies have reported that PDCoV works against the RIG-I-like receptor signaling pathway and Jak/STAT signaling pathway, which are located upstream and downstream of *I-IFN* respectively, in order to escape the host cell innate immune response [20, 21, 22]. However, the other innate immune-related signaling pathways that are affected by PDCoV infection against the host cell innate immune response discovered in our present study also deserve attention and should be explored further in the future.

Since host cell endogenous miRNAs are important contributors to the modulation of cell innate immunity by coronaviruses [23–29], porcine miRNAs belonging to PK-15 cells that may modulate the 4 important PRRs-mediated innate immune signalings by targeting unique or multiple genes involved in these signalings were predicted in present study, which provides the foundation for a new research field concerning the investigation of the PDCoV infection mechanism. However, whether these miRNAs are required for PDCoV-induced innate immune modulation by regulating the potential target genes listed in the results (Tables 6 and 7) still need to be further explore.

## Conclusions

The preliminary results described in this report explore the innate immune-associated genes and signaling pathways affected by PDCoV infection, which provide the potential host cell endogenous miRNAs that may contribute to the modulation of innate immune response induced by PDCoV infection through the regulation of innate immune genes and pathways. This study will lead to further research studies into the mechanism of PDCoV infection as well as the prevention of PDCoV-induced swine diarrheal disease.

## Supporting information

S1 Table Primers used for qRT-PCR experiments. (XLSX)

S2 Table Differentially expressed genes in INF_24h versus NC. (XLSX)

S3 Table Differentially expressed genes in INF_36h versus INF_24h. (XLSX)

S4 Table GO enrichment results of INF_24h versus NC. (XLSX)

S5 Table GO enrichment results of INF_36h versus INF_24h. (XLSX)

## References

[1] Ma Y, Zhang Y, Liang X, Lou F, Oglesbee M, Krakowka S, et al Origin, evolution, and virulence of porcine deltacoronaviruses in the United States. M Bio. 2015;6(2):e00064–15. 10.1128/mBio.00064-15 25759498 PMC4453528

[2] Hu H, Jung K, Vlasova AN, Saif LJ. Experimental infection of gnotobiotic pigs with the cell-culture-adapted porcine deltacoronavirus strain OH-FD22. Arch Virol. 2016;161:3421–34. 10.1007/s00705-016-3056-8 27619798 PMC7087098

[3] Woo PC, Lau SK, Lam CS, Lau CC, Tsang AK, Lau JH, et al Discovery of seven novel mammalian and avian coronaviruses in the genus deltacoronavirus supports bat coronaviruses as the gene source of alphacoronavirus and betacoronavirus and avian coronaviruses as the gene source of gammacoronavirus and deltacoronavirus. J Virol. 2012;86(7):3995–4008. 10.1128/JVI.06540-11 22278237 PMC3302495

[4] Wang L, Byrum B, Zhang Y. Detection and genetic characterization of a deltacoronavirus in pigs in the United States, Ohio, USA, 2014. Emerg Infect Dis. 2014;20(7):1227–30. 10.3201/eid2007.140296 24964136 PMC4073853

[5] Wang L, Byrum B, Zhang Y. Porcine coronavirus HKU15 detected in 9 US states, 2014. Emerg Infect Dis. 2014;20:1594–5. 10.3201/eid2009.140756 25153521 PMC4178395

[6] Ajayi T, Dara R, Misener M, Pasma T, Moser L, Poljak Z. Herd-level prevalence and incidence of porcine epidemic diarrhoea virus (PEDV) and porcine deltacoronavirus (PDCoV) in swine herds in Ontario, Canada. Transbound Emerg Dis. 2018;65(5):1197–207. 10.1111/tbed.12858 29607611 PMC7169835

[7] Lee S, Lee C. Complete genome characterization of Korean porcine deltacoronavirus strain KOR/KNU14-04/2014. Genome Announc. 2014;2(6):e01191–214. 10.1128/genomeA.01191-14 25428966 PMC4246158

[8] Janetanakit T, Lumyai M, Bunpapong N, Boonyapisitsopa S, Chaiyawong S, Nonthabenjawan N, et al Porcine deltacoronavirus, Thailand, 2015. Emerg Infect Dis. 2016;22(4):757–59. 10.3201/eid2204.151852 26982324 PMC4806967

[9] Wang YW, Yue H, Fang W, Huang YW. Complete genome sequence of porcine deltacoronavirus strain CH/Sichuan/S27/2012 from mainland China. Genome Announc. 2015;3(5):e00945–15. 10.1128/genomeA.00945-15 26337879 PMC4559728

[10] Masuda T, Tsuchiaka S, Ashiba T, Yamasato H, Fukunari K, Omatsu T, et al Development of one-step real-time reverse transcriptase-PCR-based assays for the rapid and simultaneous detection of four viruses causing porcine diarrhea. Jpn J Vet Res. 2016;64(1):5–14. 27348884

[11] Thachil A, Gerber PF, Xiao CT, Huang YW, Opriessnig T. Development and application of an ELISA for the detection of porcine deltacoronavirus IgG antibodies. PLoS One. 2015;10(4):e0124363 10.1371/journal.pone.0124363 25881086 PMC4399883

[12] Zhang Q, Yoo D. Immune evasion of porcine enteric coronaviruses and viral modulation of antiviral innate signaling. Virus Res. 2016;226:128–41. 10.1016/j.virusres.2016.05.015 27212682 PMC7111337

[13] Zhang Q, Ma J, Yoo D. Inhibition of NF-κB activity by the porcine epidemic diarrhea virus nonstructural protein 1 for innate immune evasion. Viroloy. 2017;510:111–1126. 10.1016/j.virol.2017.07.009 28715653 PMC7111422

[14] J Horner SM. Activation and evasion of antiviral innate immunity by hepatitis C virus. Mol Biol. 2014;426(6):1198–209. 10.1016/j.jmb.2013.10.032 24184198 PMC4431647

[15] Kopecky-Bromberg SA, Martínez-Sobrido L, Frieman M, Baric RA, Palese P. Severe acute respiratory syndrome coronavirus open reading frame (ORF) 3b, ORF 6, and nucleocapsid proteins function as interferon antagonists. J Virol. 2007;81(2):548–57. 10.1128/JVI.01782-06 17108024 PMC1797484

[16] Hu Y, Li W, Gao T, Cui Y, Jin Y, Li P, et al The severe acute respiratory syndrome coronavirus nucleocapsid inhibits type I interferon production by interfering with TRIM25-mediated RIG-I ubiquitination. J Virol. 2017;91(8):e02143–16. 10.1128/JVI.02143-16 28148787 PMC5375661

[17] Ding Z, Fang L, Yuan S, Zhao L, Wang X, Long S, et al The nucleocapsid proteins of mouse hepatitis virus and severe acute respiratory syndrome coronavirus share the same IFN-β antagonizing mechanism: attenuation of PACT-mediated RIG-I/ MDA5 activation. Oncotarget. 2017;8(30):49655–49670. 10.18632/oncotarget.17912 28591694 PMC5564796

[18] Yu L, Zhang X, Wu T, Su J, Wang Y, Wang Y, et al Avian infectious bronchitis virus disrupts the melanoma differentiation associated gene 5 (MDA5) signaling pathway by cleavage of the adaptor protein MAVS. BMC Vet Res. 2017;13(1):332 10.1186/s12917-017-1253-7 29132350 PMC5683607

[19] Luo J, Fang L, Dong N, Fang P, Ding Z, Wang D, et al Porcine deltacoronavirus (PDCoV) infection suppresses RIG-I-mediated interferon-β production. 2016;495:10–7. 10.1016/j.virol.2016.04.025 27152478 PMC7111668

[20] Zhu X, Wang D, Zhou J, Pan T, Chen J, Yang Y, et al Porcine deltacoronavirus nsp5 antagonizes type I interferon signaling by cleaving STAT2. J Virol. 2017;91(10):e00003–17. 10.1128/JVI.00003-17 28250121 PMC5411617

[21] Zhu X, Fang L, Wang D, Yang Y, Chen J, Ye X, et al Porcine deltacoronavirus nsp5 inhibits interferon-β production through the cleavage of NEMO. Virology. 2017;502:33–38. 10.1016/j.virol.2016.12.005 27984784 PMC7111669

[22] Fang P, Fang L, Ren J, Hong Y, Liu X, Zhao Y, et al Porcine deltacoronavirus accessory protein NS6 antagonizes interferon beta production by interfering with the binding of RIG-I/MDA5 to double-stranded RNA. J Virol. 2018;92(15):e00712–18. 10.1128/JVI.00712-18 29769346 PMC6052322

[23] Huang J, Lang Q, Li X, Xu Z, Zhu L, Zhou Y. MicroRNA Expression Profiles of Porcine Kidney 15 Cell Line Infected with Porcine Epidemic Diahorrea Virus. Chinese Journal of Virology. 2016;32(4):465–71. (in Chinese) 29995369

[24] Mallick B, Ghosh Z, Chakrabarti J. MicroRNome analysis unravels the molecular basis of SARS infection in bronchoalveolar stem cells. PLoS One. 2009;4(11):e7837 10.1371/journal.pone.0007837 19915717 PMC2773932

[25] Devhare PB, Steele R, Di Bisceglie AM, Kaplan DE, Ray RB. Differential Expression of MicroRNAs in Hepatitis C Virus-Mediated Liver Disease Between African Americans and Caucasians: Implications for Racial Health Disparities. Gene Expr. 2017;17(2):89–98. 10.3727/105221616X693594 27765085 PMC8751126

[26] Liu X, Zhu L, Liao S, Xu Z, Zhou Y. The porcine microRNA transcriptome response to transmissible gastroenteritis virus infection. PLoS One. 2015;10(3):e0120377 10.1371/journal.pone.0120377 25781021 PMC4363316

[27] Zheng H, Xu L, Liu Y, Li C, Zhang L, Wang T, et al MicroRNA-221-5p Inhibits Porcine Epidemic Diarrhea Virus Replication by Targeting Genomic Viral RNA and Activating the NF-κB Pathway. Int J Mol Sci. 2018;19(11). pii: E3381 10.3390/ijms19113381 30380612 PMC6274926

[28] Ma Y, Wang C, Xue M, Fu F, Zhang X, Li L, et al Coronavirus TGEV evades the type I interferon response through IRE1α-mediated manipulation of the miR-30a-5p/SOCS1/3 Axis. J Virol. 2018;92(22):e00728–18. 10.1128/JVI.00728-18 30185587 PMC6206482

[29] Bhanja Chowdhury J, Shrivastava S, Steele R, Di Bisceglie AM, Ray R, Ray RB. Hepatitis C virus infection modulates expression of interferon stimulatory gene IFITM1 by upregulating miR-130A. J Virol. 2012;86(18):10221–5. 10.1128/JVI.00882-12 22787204 PMC3446586

[30] Zheng L, Li XL, Yan MH, Ren WK, Zhang L, Lu C, et al Isolation, identification and biological characteristics analysis of porcine deltacoronavirus TJ_1_[J]. China Animal Husbandry & Veterinary Medicine. 2018;45(1):219–224. (in Chinese)

[31] Anders S, Huber W. Differential expression analysis for sequence count data. Genome Biol. 2010;11(10):R106 10.1186/gb-2010-11-10-r106 20979621 PMC3218662

[32] Trapnell C, Williams BA, Pertea G, Mortazavi A, Kwan G, van Baren MJ, et al Transcript assembly and quantification by RNA-Seq reveals unannotated transcripts and isoform switching during cell differentiation. Nat Biotechnol. 2010;28(5):511–5. 10.1038/nbt.1621 20436464 PMC3146043

[33] Thompson HW, Mera R, Prasad C. A description of the appropriate use of student’s t-test. Nutr Neurosci. 1998;1(2):165–72. 10.1080/1028415X.1998.11747226 27406022

[34] Mair KH, Sedlak C, Käser T, Pasternak A, Levast B, Gerner W, et al The porcine innate immune system: An update. Dev Comp Immunol. 2014;45(2):321–43. 10.1016/j.dci.2014.03.022 24709051 PMC7103209

[35] Zhang H, Liu Q, Su W, Wang J, Sun Y, Zhang J, et al Genome-wide analysis of differentially expressed genes and the modulation of PEDV infection in Vero E6 cells. Microb Pathog. 2018;117:247–54. 10.1016/j.micpath.2018.02.004 29408315 PMC7125602

[36] Hu H, Jung K, Vlasova AN, Chepngeno J, Lu Z, Wang Q, et al Isolation and characterization of porcine deltacoronavirus from pigs with diarrhea in the United States. J Clin Microbiol. 2015;53(5):1537–48. 10.1128/JCM.00031-15 25740769 PMC4400786

[37] Zhang J, Chen J, Shi D, Shi H, Zhang X, Liu J, et al Porcine deltacoronavirus enters cells via two pathways: A protease-mediated one at the cell surface and another facilitated by cathepsins in the endosome. Journal of biological chemistry. J Biol Chem. 2019;294(25):9830–9843. 10.1074/jbc.RA119.007779 31068417 PMC6597833

